# Ubiquitin-specific protease 29 attenuates hepatic ischemia-reperfusion injury by mediating TGF-β-activated kinase 1 deubiquitination

**DOI:** 10.3389/fimmu.2023.1167667

**Published:** 2023-05-26

**Authors:** Zhongbao Chen, Fengjiao Hu, Yalong Zhang, Long Zhang, Tianyu Wang, Chenyang Kong, Haochong Hu, Jiayu Guo, Qi Chen, Bo Yu, Yiting Liu, Jilin Zou, Jiangqiao Zhou, Tao Qiu

**Affiliations:** ^1^Department of Organ Transplantation, Renmin Hospital, Wuhan University, Wuhan, China; ^2^Department of Urology, Renmin Hospital, Wuhan University, Wuhan, China; ^3^Medical Science Research Centre, Zhongnan Hospital, Wuhan University, Wuhan, China

**Keywords:** Usp29, TAK1, deubiquitination, hepatic ischemia-reperfusion injury, inflammation, apoptosis, phosphorylation

## Abstract

**Background and aims:**

In the course of clinical practice, hepatic ischemia/reperfusion (I/R) injury is a prevalent pathophysiological event and is caused by a combination of complex factors that involve multiple signaling pathways such as MAPK and NF-κB. USP29 is a deubiquitinating enzyme important during the development of tumors, neurological diseases, and viral immunity. However, it is unknown how USP29 contributes to hepatic I/R injury.

**Methods and results:**

We systematically investigated the role of the USP29/TAK1-JNK/p38 signaling pathway in hepatic I/R injury. We first found reduced USP29 expression in both mouse hepatic I/R injury and the primary hepatocyte hypoxia-reoxygenation (H/R) models. We established USP29 full knockout mice (USP29-KO) and hepatocyte-specific USP29 transgenic mice (USP29-HTG), and we found that USP29 knockout significantly exacerbates the inflammatory infiltration and injury processes during hepatic I/R injury, whereas USP29 overexpression alleviates liver injury by decreasing the inflammatory response and inhibiting apoptosis. Mechanistically, RNA sequencing results showed the effects of USP29 on the MAPK pathway, and further studies revealed that USP29 interacts with TAK1 and inhibits its k63-linked polyubiquitination, thereby preventing the activation of TAK1 and its downstream signaling pathways. Consistently, 5z-7-Oxozeaneol, an inhibitor of TAK1, blocked the detrimental effects of USP29 knockout on H/R-induced hepatocyte injury, further confirming that USP29 plays a regulatory role in hepatic I/R injury by targeting TAK1.

**Conclusion:**

Our findings imply that USP29 is a therapeutic target with promise for the management of hepatic I/R injury via TAK1-JNK/p38 pathway-dependent processes.

## Introduction

Hepatic ischemia/reperfusion (I/R) injury is a severe inflammatory response driven by innate immunity (1) during liver transplantation, partial hepatectomy, hypovolemic shock, or toxic liver damage and it can lead to liver dysfunction or failure, and even to death (2). An inflammatory cascade and hepatocyte apoptosis are prominent hepatic I/R injury features. However, no effective clinical treatment for hepatic I/R injury exists, and specific therapeutic measures or potent drugs are lacking. Therefore, clarifying the I/R injury pathogenesis is an urgent need to be able to discover new therapeutic targets.

I/R injury is a complex pathophysiological mechanism linked to oxidative stress, calcium overload, abnormal mitochondrial structure and function, Kupffer cell and neutrophil activation, inflammatory factor release, and apoptosis, and it has not yet been fully elucidated (3–5). These mechanisms are initiated during the course of liver ischemia and are further aggravated after blood reperfusion. Ischemic/hypoxic hepatocytes undergo apoptosis and necrosis and then release dangerous pattern-signaling molecules such as Toll-like receptor activators that promote hepatocyte inflammation and activate immune cells in the liver to release chemotaxis factors, thereby recruiting peripheral inflammatory cells, leading to an inflammatory waterfall cascade that further causes liver tissue injury (6, 7). This mutual positive feedback inflammation-apoptosis loop ultimately leads to liver tissue damage.

Ubiquitin-Specific Protease 29 (USP29) is a ubiquitin-proteasome family member, first identified in the year 2000 and located in the PEG3 (Progression-elevated gene-3) gene imprint on mouse chromosome 7 and human chromosome 19, where it is aligned head-to-head with the PEG3 gene (8). The main USP29 structural domain lies in its C-terminal USP domain, an essential player in a variety of disease processes through the deubiquitination of regulatory substrates. For example, USP29 acts as a traumatic spinal cord injury protector by deubiquitinating NRF2 (Nuclear erythroid 2-related factor 2), thereby stabilizing NRF2 function and regulating microglia/macrophage polarization (9). In tumor studies of liver, stomach, and colon cancers, USP29 is also essential during tumorigenesis and chemotherapy resistance by stabilizing the expression and function of various substrate proteins, such as Cdc25A (Cell division cycle 25A), Snail, MYC, and HIF1α, through deubiquitination, thereby affecting the proliferation, invasion, and tumor cell migration (10–15). However, the role of USP29 in hepatic I/R injury remains unclear.

In this study, we show that USP29 expression was significantly downregulated during hepatic I/R. *In vitro* and *in vivo* studies suggest that USP29 plays a protective role during hepatic I/R by attenuating the inflammatory response and inhibiting apoptosis. Mechanistically, USP29 reduces the sterile inflammation and hepatocellular damage by deubiquitinating TAK1 and preventing its phosphorylation, thereby suppressing the activation of its downstream JNK/p38 pathways. In conclusion, our study reveals a mechanism by which USP29 regulates I/R liver injury, indispensable knowledge for generating new treatments.

## Materials and methods

### Human liver samples

Human liver specimens were obtained from liver transplantation donor patients in the Renmin Hospital of Wuhan University (Wuhan, China). Informed consent was obtained from all patients or their families to sign a consent form for the use of clinical specimens. Pre-ischemic liver specimens (Pre group) were taken prior to the closure of the portal vein, and post-reperfusion liver specimens (Post group) were taken approximately two hours after portal vein reperfusion. All operations that involved human sample collection and application were approved by the ethics committee of Renmin Hospital of Wuhan University.

### Animals and handling

Male C57BL/6 mice aged 8 weeks were purchased from Beijing Vital River Laboratory Animal Technology Co., Ltd. (Beijing, China) and housed in the Experimental Animal Center of the Renmin Hospital of Wuhan University. The mice were housed in individual cages at room temperature with a 24-hour light cycle, unrestricted access to food and water, and a free feed in a serene atmosphere with a humidity level of 55% and a temperature of about 21°C. All animal husbandry and experimental operations followed the laboratory management and ethical requirements of the Renmin Hospital of Wuhan University. We established a mouse model of 70% hepatic I/R injury using a published method (16)and collected liver and serum specimens for experiments. USP29 knockout (USP29-KO) and hepatocyte-specific USP29 transgenic mice (USP29-HTG) mice were generated as detailed in the **Supporting Information.**

### Histological analysis

Mouse liver tissues were fixed with 10% formalin for 72h, routinely dehydrated and paraffin-embedded, and then serially sectioned to a 5 μm thickness. After tissue dewaxing, standard hematoxylin(G1004, Servicebio, Wuhan, China) and eosin (BA-4024, BaSO, Zhuhai, China)(H&E) staining showed areas of liver necrosis areas, We used CD11b immunofluorescence (IF) to label inflammatory cell infiltration and terminal deoxynucleotidyl transferase labeling (TUNEL) to show the proportion of apoptotic cells in the liver. The detailed method steps are shown in the Supporting Information. We used optical microscopy Image-Pro Plus 6.1 software to observe and capture images.

### RNA sequencing and analysis

We extracted total RNA from WT and USP29-KO mouse biopsies to profile the gene expression differences. After assessing the quality of the RNA, we used the MGIEasy RNA library kit to construct cDNA libraries. Gene expression profiling was performed using a BGISEQ-500 instrument. After removing low-quality reads, clean reads were mapped to the reference genome sequence (MM10) using HisAT2 software (version 2.1.0). Stringtie (version 1.3.3b) was used to identify the number of fragments per kilobase per million (FPKM) and to obtain specific gene expression information. Differential expression analysis of the gene expression matrix was performed by DESeq2(version 1.2.10). Obtain and visualize differentially expressed genes as a fold change greater than 1.5 and P values corrected for multiple comparisons less than 0.05. RNA-seq data were analyzed using GSEA 4.1.0 software. False discovery rate (FDR) was used for the statistical significance assessment of NES. Gene sets with FDR<0.25 were considered statistically significant. FDR was controlled by adjusting the p values using the Benjamini-Hochberg algorithm. Gene-enrichment and functional annotation analysis and pathway analysis for significant gene lists were performed based on the Kyoto Encyclopedia of Genes and Genomes (KEGG) pathway. The Fisher exact test was used to identify KEGG pathways that were significantly enriched for differential genes and pathways with p<0.05 were considered significantly enriched.

### Cell culture and treatment

L02 and HEK293 cells were purchased from the Typical Culture Collection Committee Cell Bank, Chinese Academy of Sciences (Shanghai, China) and cultured in DMEM (BC-M-005, Bio-Channel, Nanjing, China) medium supplemented with 10% fetal bovine serum (F05-001-B160216; Bio-One Biotechnology, Guangzhou, China) and 1% penicillin-streptomycin (15140-122; Gibco by Invitrogen). Cells proliferated to an abundance of 80%-90% and were digested by trypsin and subcultured. Mouse primary hepatocytes were isolated from the liver of 6-8 week-old male mice by collagenous IV digestion (17), and cultured in DMEM medium containing serum and double antibodies in mouse tail gel-coated culture dishes. The cells were maintained at 37°C with 5% CO2 in an incubator. The hypoxia/reoxygenation (H/R) model of mouse primary hepatocytes was established according to the steps in the **Supporting Information.**

### Plasmids and stable cell-line construction

The full-length CDS sequences of *USP29*, *TAK1*, and *ASK1* were amplified from human cDNA, and the full-length CDS of *USP29*, *TAK1*, and *ASK1* were concatenated into pcDNA5-flag, pcDNA5-HA, and *GST-HA* vectors by In-fusion recombination to obtain *USP29*, *TAK1* and *ASK1* full-length overexpression plasmids (Flag tag, HA tag, GST-HA tag). We also designed primers to construct truncated plasmids for Flag-*USP29* (1-283, 284-922) and Flag-*TAK1* (1-480, 481-579).All the primers are listed in the **Supplementary Table 2.**

### Western blotting analysis

We extracted Proteins from ischemic liver tissue or cell lysates and determined their concentration using the BCA (23225, Thermo) method. Protein samples of the same mass were added to the one-quarter volume of 5x loading buffer, boiling water bath for 10 minutes, and the supernatant was separated by SDS-PAGE. After electrophoresis, the proteins were transferred onto 0.45 μm PVDF (IPVH00010, Millipore) membranes. After transfer, PVDF membranes were blocked with 5% skim milk powder containing 0.1% Tween 20 (TBST) for about 1 hour at room temperature. We washed PVDF membranes twice with TBST for 5 min each time and then incubated each membrane with primary antibody at 4°C overnight. The PVDF membranes were washed three times with TBST for 10 min each time and incubated with peroxidase-coupled secondary antibody (Cell Signaling Technology, Danvers, MA) for 1 h at room temperature. The PVDF membranes were then washed again with TBST three times for 10 min each time. We collected the final results on the basics of signals from the Burroughs Gel Imaging System (ChemiDoc XRS, +). **Supplementary Table 4** lists the antibodies used.

### Real-time quantitative PCR

TRIzol reagent (T9424, Sigma) was used to extract total RNA from collected mouse liver tissue and cultured mouse primary hepatocytes, as directed by the kit instructions. Total RNA was reverse transcribed to cDNA using the Vazyme reverse transcription kit. real-time PCR was performed using the standard SYBR Green PCR kit (Q111-02, vazyme) and LightCycler 480 (Roche). We used β-actin as an internal reference to calculate the relative expression.

### Statistical analysis

We used the Statistical Product and Service Solutions (SPSS; version 26.0; IBM, Armonk, NY, USA) software and Graphpad Prism (GraphPad Software, version 8.4;San Diego, California) software were used for data analysis and statistical charting. All measures are expressed as means ± SD. We applied unpaired two-tailed *t*-tests on normally distributed data for analyses between two groups. We used One-way ANOVA was used for multiple comparisons, Bonferroni analysis was used for data consistent with chi-square, and Tamhane’s T2 analysis was used for data with heteroskedasticity. For data sets with skewed distributions, We performed nonparametric statistical analyses for both groups using the Mann- Whitney U test. We considered all differences with a *p* < 0.05 as statistically significant.

## Results

### USP29 expression is downregulated in both *in vivo* and ex vivo hepatic I/R injury modes

**Figure 1:**
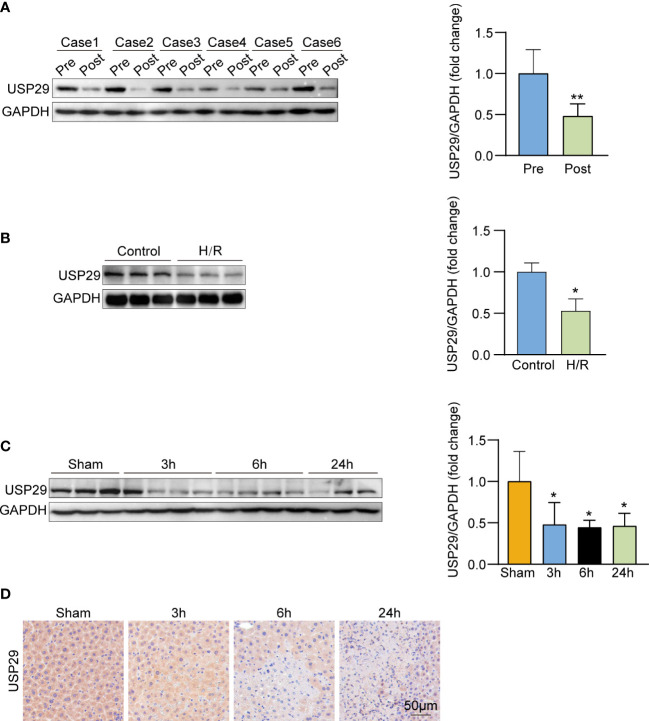
USP29 expression is down-regulated in vivo and in vitro hepatic ischemia/reperfusion (I/R) injury models. **(A)** USP29 protein expression in pre-ischemic and after reperfusion clinical human liver graft biopsies (n = 6 per group). **(B)** Western blot detection of USP29 protein expression in primary hepatocytes with hypoxia/reperfusion (H/R) treatment(n = 6 per group). **(C)** Western blot detection of USP29 protein expression in liver from mice subjected to sham or ischemia treatment for 1 hour, followed by reperfusion for 3, 6, and 24 h (n = 6 per time point). GAPDH served as the loading control. **(D)** Representative immunohistochemical staining of USP29 expression in liver lobes from WT mice subjected to sham or ischemia treatment for 1 hour, followed by reperfusion for 3, 6, and 24 h (n = 6 per time point). Scale bar, 50 μm. All data are expressed as means ± SDs. *p <0.05; **p <0.01. GAPDH, glyceraldehyde-3-phosphate dehydrogenase.

We first examined the USP29 expression in the liver biopsies before and after liver transplantations of patients who had undergone the clinical treatment. Notably, the USP29 protein expression was significantly lower in liver samples after reperfusion than in those before ischemia (**Figure 1A**). Next, we explored USP29 expression changes in the mouse primary hepatocyte H/R model. USP29 protein expression was markedly downregulated in primary hepatocytes 6h after the H/R stimulation challenge (**Figure 1B**). We next explored whether the *in vivo* animal model results were consistent with the *in vitro* results. To obtain the optimal conditions, we examined the USP29 expression changes at the protein level after 1 h of ischemia and 3, 6, and 24 h of reperfusion. We found that USP29 protein expression levels decreased significantly after reperfusion, but no statistical differences between the USP29 expression levels at 3, 6, and 24 h of reperfusion (**Figure 1C**). Therefore, we selected the 1h after ischemia and 3h after reperfusion time points as the challenge conditions for the subsequent I/R injury mouse liver model experiments. In addition, IHC staining results confirmed the significantly downregulated USP29 expression in the hepatic I/R injury samples compared with the expression in the liver sham-operated samples (**Figure 1D**). These findings suggest that USP29 expression is associated with hepatic I/R injury and that USP29 may play an important role in the development of hepatic I/R.

### USP29 attenuates H/R-stimulated hepatocyte inflammation and apoptosis

**Figure 2:**
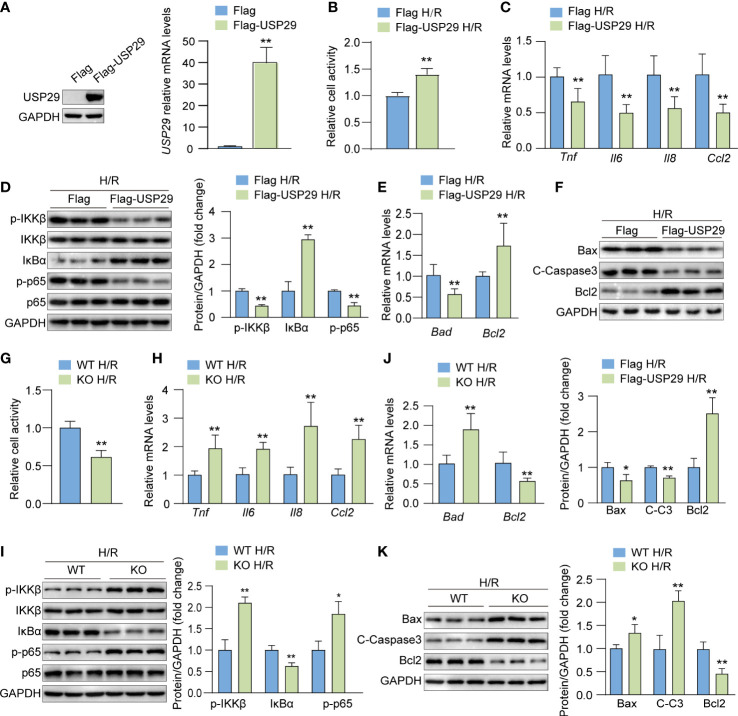
USP29 suppresses inflammation and apoptosis in hypoxia/reoxygenation (H/R)-treated hepatocytes. **(A)** USP29 protein (left) and mRNA (right) levels in primary hepatocytes transfected with control (Flag) and USP29 overexpressing (Flag-USP29) adenoviruses. **(B)** Cell viability in the indicated groups treated with H/R. **(C)** mRNA levels of proinflammatory factors (Tnf, Il6, Il8, and Ccl2) in the indicated groups treated with H/R. **(D)** Protein levels of the NF-κB signaling components in the indicated groups treated with H/R as detected by Western blotting. **(E)** mRNA levels of apoptosis-associated genes in the indicated groups treated with H/R. **(F)** Apoptosis-associated protein levels in the indicated groups treated with H/R. **(G)** Cell viability in H/R-treated hepatocytes isolated from USP29-KO and WT mice. **(H)** mRNA levels of proinflammatory factors (Tnf, Il6, Il8 and Ccl2) in the indicated groups treated with H/R. **(I)** NF-κB signaling component protein levels in the indicated groups treated with H/R. **(J)** mRNA levels of apoptosis-associated genes in the indicated groups treated with H/R. **(K)** Apoptosis-associated protein levels in the indicated groups treated with H/R. GAPDH served as the loading control in **(A, D, F, I, K)**. All data are expressed as means ± SDs (Data are representative of three independent experiments). *p < 0.05; **p < 0.01. ALT, alanine transaminase; AST, aspartate transaminase; C-Caspase3, cleaved caspase-3; Ccl2, C-C motif chemokine ligand 2; Ccl5, C-C motif chemokine ligand 5; GAPDH, glyceraldehyde-3-phosphate dehydrogenase; IL-6, interleukin-6; IL-8, interleukin-8; IκBα, inhibitory κB α; IKKβ, IκB kinase β; PHF, per high field; p-IKKβ, phosphorylated IKKβ; p-p65, phosphorylated p65; TNF, tumor necrosis factor.

To investigate the effect of USP29 on hepatocyte apoptosis and inflammatory response in response to H/R stimulation *in vitro*, we overexpressed USP29 in primary hepatocytes by adenovirus infection and obtained USP29 knockout primary hepatocytes by isolating the livers of USP29-KO mice. We confirmed the USP29 overexpression by Western blot analysis and qPCR (**Figure 2A**). We examined the cell activity after the H/R challenge with a CCK-8 assay and found that the cell viability of the Flag-USP29 group was significantly higher than that of control cells (**Figure 2B**), which indicates that USP29 overexpression enhanced the cells’ tolerance to the H/R challenge. We also found that USP29 overexpression in hepatocytes significantly reduced the expression levels of inflammatory factors (*Tnf*, *Il6*, *Il8* and *Ccl2*) and inhibited the activation of the NF-κB signaling pathway (**Figures 2C, D**). In addition, after the H/R challenge, USP29 overexpression in hepatocytes suppressed the expression of pro-apoptotic-related molecules (*Bad*, *Bax*, and *C-caspase 3*) and promoted the expression of Bcl-2 when compared to those expressions in the control cells (**Figures 2E, F**). In contrast, USP29 knockout exacerbated the damage in H/R-treated hepatocytes, we found a significant decrease in cell viability compared to control (**Figure 2G**), elevated expression of pro-inflammatory-related factors (*Tnf*, *Il6*, *Il8*, and *Ccl2*) (**Figure 2H**), activation of NF-κB signaling pathway (**Figure 2I**), increased expression of pro-apoptotic factors (*Bad*, *Bax* and *C-caspase 3*), and decreased the expression of apoptosis suppressor *Bcl2* (**Figures 2J, K**) in these cells when compared to the findings in control cells. These findings clearly indicate that USP29 inhibited H/R-induced inflammation and apoptosis in primary mouse hepatocytes.

### USP29 deficiency exacerbates hepatic injury, inflammation response, and apoptosis

**Figure 3:**
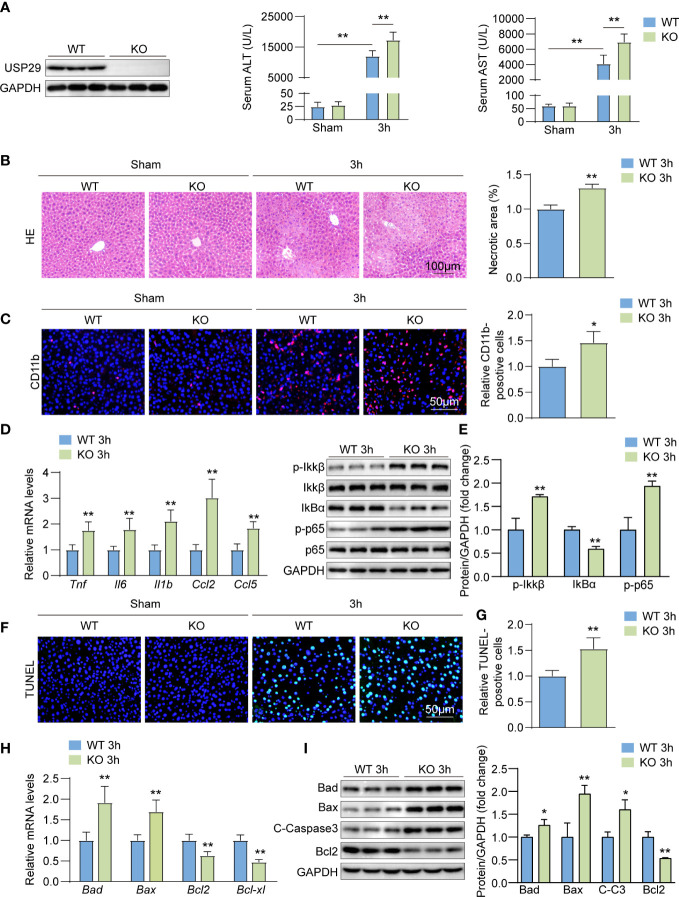
USP29 knockdown exacerbates liver ischemia/reperfusion (I/R) injury by promoting inflammation and apoptosis. **(A)** Western blot analysis of USP29 levels in livers from USP29-KO and WT mice (n = 3 per group). **(B)** Serum ALT and AST levels in USP29-KO and WT mice 3 hours after reperfusion or sham treatment (n = 9 per group). **(C)** Representative liver H&E staining image (left) and quantification (right) from USP29-KO and WT mice 3 hours after reperfusion or sham treatment (n= 6 per group). Scale bar, 100 μm. **(D)** Representative CD11b immunofluorescence staining and quantification in livers of USP29-KO and WT mice 3 hours after reperfusion or sham treatment (n = 4 per group). Scale bar, 50 μm. **(E)** mRNA levels of Tnf, Il6, Il1b,Ccl2 and Ccl5 in liver tissues from USP29-KO and WT mice 3 hours after reperfusion treatment (n = 5 mice per group). **(F)** Western blotting detection of NF-κB signaling components in liver tissues from USP29-KO and WT mice 3 hours after reperfusion treatment (n= 3 per group). **(G)** Representative TUNEL-positive apoptotic cell images and quantification in liver tissues from USP29-KO and WT mice 3 hours after reperfusion or sham treatment (n = 4 per group). Scale bar, 50 μm. **(H)** mRNA levels of apoptosis-associated genes (Bad, Bax, Bcl2, Bcl-xl) in liver tissues from USP29-KO and WT mice 3 hours after reperfusion treatment (n = 5 per group). **(I)** Western blot detection of apoptosis-associated proteins (Bad, Bax, C-caspase3, Bcl2) levels in liver tissue from USP29-KO and WT mice 3 hours after reperfusion treatment (n = 3 per group). GAPDH served as the loading control. All data are shown as means ± SDs. *p < 0.05; **p < 0.01. ALT, alanine transaminase; AST, aspartate transaminase; C-Caspase3, cleaved caspase-3; Ccl2, C-C motif chemokine ligand 2; Ccl5, C-C motif chemokine ligand 5; GAPDH, glyceraldehyde-3-phosphate dehydrogenase; IL-1β, interleukin-1β; IL-6, interleukin-6; IκBα, inhibitory κB α; IKKβ, IκB kinase β; p-IKKβ, phosphorylated IKKβ; p-p65, phosphorylated p65; TNF, Tumor necrosis factor.

To further evaluate the role of USP29 during hepatic I/R injury, we constructed USP29 full knockout mice and used western blotting to confirm the deletion of USP29 in the liver (**Figure 3A**). And 3h after I/R, we found the serum alanine aminotransferase (ALT) and aspartate aminotransferase (AST) levels in the USP29-KO group were significantly higher than that in the WT group (**Figure 3B**). The histological examination further showed that 3h after I/R, the necrotic areas were markedly larger in USP29-KO mice than in WT controls (**Figure 3C**). Immunofluorescence staining showed that CD11b-positive inflammatory cell infiltration was increased in the liver of WT mice 3h after hepatic I/R (**Figure 3D**). In addition, mRNA levels of pro-inflammatory cytokines and chemokines, such as tumor necrosis factor (*Tnf*), interleukin 6 (*Il6*), interleukin-1b (*IL1b*), C-C motif chemokine ligand 2 (*Ccl2*), and C-C motif chemokine ligand 5 (*Ccl5*) were also increased in the USP29-KO mice after I/R injury (**Figure 3E**). NF-κB is a classical inflammation-related signaling pathway. When subjected to external stimuli or internal environmental changes, the NF-κB pathway gets activated and can induce the expression of a series of genes, such as many adhesion factors, increased expression and release of inflammatory factors, chemokines, and some receptor molecules, leading to further amplification of the initial inflammatory signal and aggravation of the organism damage (18). NF-κB signaling pathway activation was more intense in liver tissues of I/R-treated USP29-KO mice than in WT controls (**Figure 3F**). We examined the effect of USP29 on hepatic apoptosis (another prominent hepatic I/R injury marker) using TUNEL staining. Our results showed that the number of apoptotic cells was significantly higher in USP29-KO tissues after hepatic I/R than in the control tissues (**Figure 3G**). Furthermore, qRT- PCR results revealed that the expression levels of pro-apoptotic genes *Bax* and *Bad* were upregulated in the USP29-KO mice after reperfusion, while anti-apoptotic genes *Bcl2* and *Bcl-xl* were downregulated (**Figure 3H**). Western blot analysis further confirmed that the expressions of pro-apoptotic molecules *Bad*, *Bax*, and cleaved caspase 3 (*C-caspase 3*) were upregulated, and that of the anti-apoptotic molecule *Bcl2* was downregulated in the liver of USP29-KO mice compared with the expressions in WT mice 3 h after I/R injury (**Figure 3I**). The above results suggest that USP29 deficiency promotes local inflammation and hepatocyte apoptosis to exacerbate hepatic I/R injury.

### USP29 overexpression inhibits hepatic injury, inflammatory response, and apoptosis

**Figure 4:**
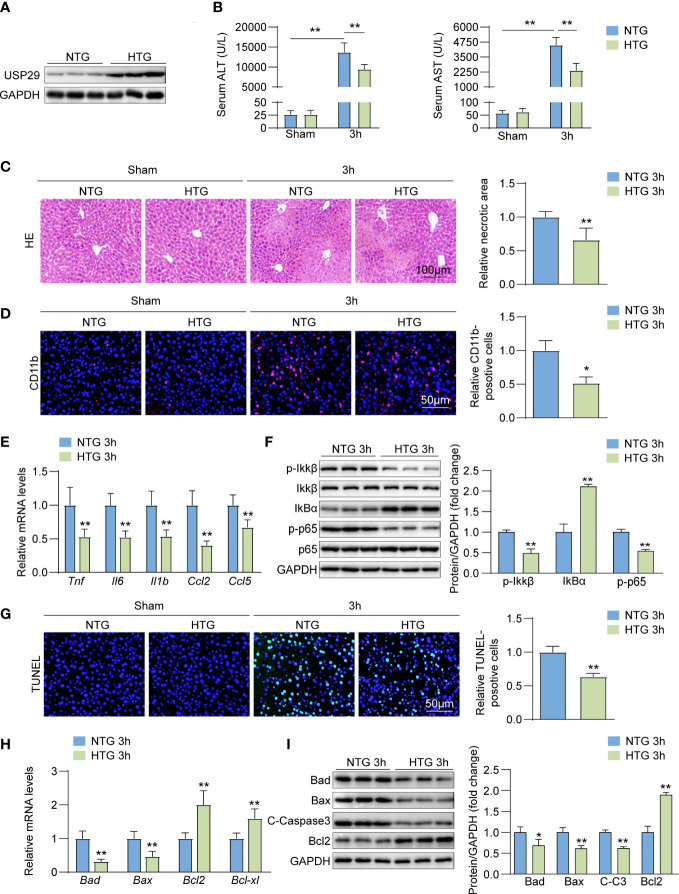
USP29 overexpression inhibits liver inflammation and apoptosis. **(A)** Western blot analysis of USP29 levels in livers from USP29-HTG and NTG mice (n = 3 per group). **(B)** Serum ALT and AST levels in USP29-HTG and NTG mice 3 hours after reperfusion or sham treatment. (n = 9 per group). **(C)** Representative liver H&E staining image (left) and quantification (right) from USP29-HTG and NTG mice 3 hours after reperfusion or sham treatment (n=6 per group). Scale bar, 100 μm. **(D)** Representative CD11b immunofluorescence staining and quantification in livers of USP29-HTG and NTG mice 3 hours after reperfusion or sham treatment (n = 4 per group). Scale bar, 50 μm. **(E)** mRNA levels of Tnf, Il6, Il1b,Ccl2 and Ccl5 in liver tissues from USP29-HTG and NTG mice 3 hours after reperfusion treatment. (n = 5 mice per group). **(F)** Western blotting detection of NF-κB signaling components in liver tissue from USP29-HTG and NTG mice 3 hours after reperfusion treatment (n = 3 per group). **(G)** Representative TUNEL-positive apoptotic cell images and quantification in liver tissues from USP29-HTG and NTG mice 3 hours after reperfusion or sham treatment (n = 4 per group). Scale bar, 50 μm. **(H)** mRNA levels of apoptosis-associated genes (Bad, Bax, Bcl2, Bcl-xl) in liver tissues from USP29-HTG and NTG mice 3 hours after reperfusion treatment (n = 5 per group). **(I)** Western blot detection of apoptosis-associated proteins (Bad, Bax, C-caspase3, Bcl2) levels in liver tissue from USP29-HTG and NTG mice 3 hours after reperfusion treatment (n = 3 per group). GAPDH served as the loading control in (A), (F), and (I). All data are shown as means ± SDs. *p < 0.05; **p < 0.01. ALT, alanine transaminase; AST, aspartate transaminase; C-Caspase3, cleaved caspase-3; Ccl2, C-C motif chemokine ligand 2; Ccl5, C-C motif chemokine ligand 5; GAPDH, glyceraldehyde-3-phosphate dehydrogenase; IL-1b, interleukin-1b; IL-6,interleukin-6; IκBα, inhibitory κB α; IKKβ, IκB kinase β; p-IKKβ, phosphorylated IKKβ; p-p65, phosphorylated p65; TNF, Tumor necrosis factor.

We constructed a USP29 liver-specific expression in mice and confirmed the USP29 overexpression in the mouse liver by Western blot analysis (**Figure 4A**). The USP29-HTG group significantly reduced the I/R-induced elevation of ALT and AST (**Figure 4B**) and the hepatic necrotic area surface (**Figure 4C**). In addition, the rate of CD11b-positive cells in the liver of the USP29-HTG group was significantly less than that in the WT group after I/R surgery (**Figure 4D**). The mRNA levels of pro-inflammatory cytokines/chemokines such as *Tnf*, *Il6*, *Il1b*, *Ccl2*, and *Ccl5* were significantly lowered in the liver tissues of USP29-HTG mice than NTG mice (**Figure 4E**). Moreover, the NF-κB pathway activation was inhibited in the USP29-HTG mice (**Figure 4F**). TUNEL staining showed that USP29-HTG attenuated apoptosis induced by hepatic I/R injury (**Figure 4G**). In addition, q-PCR (**Figure 4H**) and Western blot (**Figure 4I**) showed down-regulation of pro-apoptotic factor expressions and elevation of apoptosis suppressor expression in USP29-HTG mice. Our findings support the hypothesis that USP29 plays a protective role in the liver.

### RNA-seq results show that USP29 regulates the MAPK signaling pathway in hepatic I/R injury

**Figure 5:**
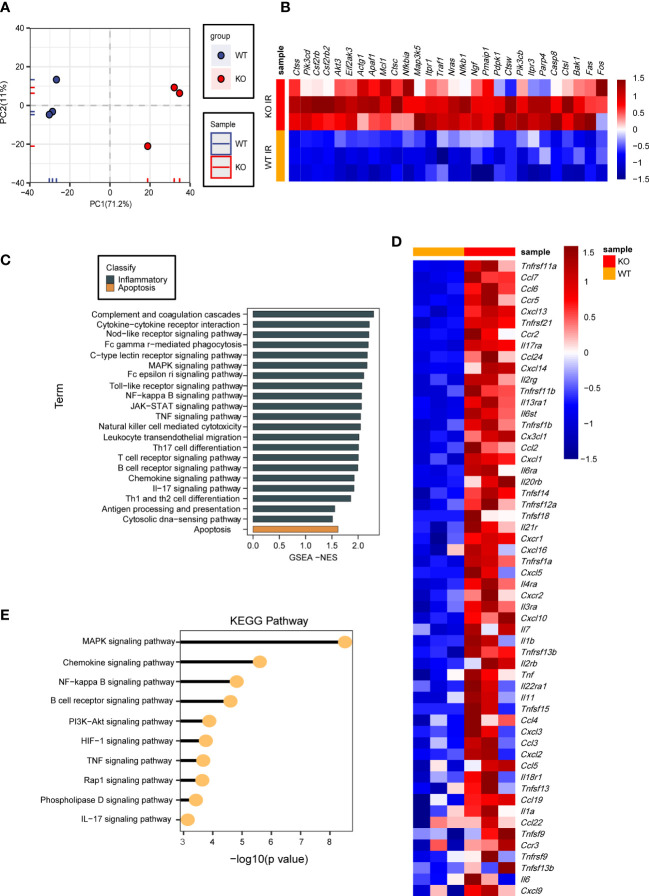
RNA-seq results showing that USP29 regulates MAPK signaling pathway in hepatic I/R injury. **(A)** PCA image showing global sample distribution profiles analyzed in the USP29-KO group relative to those in WT controls after I/R injury (n = 3 per group). **(B)** GO analysis based on leading-edge subsets of GSEA-enrichment showing the top enriched biological processes contributing to USP29 function from USP29-KO mice compared to WT mice after 3h of I/R treatment. The eighteen most significantly enriched pathways of cell death (black) and seven most significantly enriched pathways of inflammation (brown) are shown. **(C, D)** Heatmap showing the expression of apoptosis and inflammation-related gene sets with the highest leading edge subsets in the livers of USP29-KO and WT mice after 3h of I/R treatment (n = 3 per group) detected by RNA- seq analyses. **(E)** On the basis of our KEGG enrichment analysis of RNA-seq data, major biological pathways contribute to USP29 function. Actg1, actin gamma 1; Akt3, AKT serine/threonine kinase 3; Apaf1, apoptotic protease activating factor-1; Bak1, bri1-associated receptor kinase 1; Casp8, caspase-8; Ccl2, C-C motif chemokine ligand 2; Ccl3, C-C motif ligand 3; Ccl4, C-C motif ligand 4; Ccl5, C-C motif ligand 5; Ccl6, C-C motif ligand 6; Ccl7, C-C motif ligand 7; Ccl19, C-C motif ligand 19; Ccl22, C-C motif ligand 22; Ccl24, C-C motif ligand 24; Ccr2, CC chemokine receptor 2; Ccr3, CC chemokine receptor 3; Ccr5, CC chemokine receptor 5; Csf2rb, colony stimulating factor 2 receptor beta; Csf2rb2, colony stimulating factor 2 receptor beta 2 ; Ctsc, cathepsin C; Ctss, cathepsin S; Ctsw, cathepsin W; Ctsl, cathepsin L; Cxcl1, C-X-C motif ligand 1; Cxcl2, C-X-C motif ligand 2; Cxcl3, C-X-C motif ligand 3; Cxcl5, C-X-C motif ligand 5; Cxcl9, C-X-C motif ligand 9; Cxcl10, C-X-C motif ligand 10; Cxcl13, C-X-C motif ligand 13 ; Cxcl14, C-X-C motif ligand 14; Cxcl16, C-X-C motif ligand 16; Cxcr1, CXC chemokine receptor 1; Cxcr2, CXC chemokine receptor 2; Cx3cl1, CX3C chemokine ligand 1; Eif2ak3, eukaryotic translation initiation factor 2-alpha kinase 3; Fas, factor related apoptosis; Il1a, interleukin-1 alpha; Il1b, interleukin-1beta; Il2rb, interleukin-2 receptor beta; Il2rg, interleukin-2 receptor gamma chain; Il3ra, interleukin-3 receptor alpha; Il4ra, interleukin-4 receptor alpha; Il6, interleukin-6; Il6st, interleukin 6 signal transducer; Il6ra, interleukin 6 receptor alpha; Il7, interleukin-7; Il11, interleukin-11; Il13ra1, interleukin-13 receptor alpha 1; Il17ra, interleukin-17 receptor alpha; Il18r1, interleukin-18 receptor 1; Il20rb, interleukin-20 receptor beta; Il21r, interleukin-21 receptor; Il22ra1, interleukin-22 receptor alpha 1; Itpr1, inositol 1,4,5-trisphosphate receptor 1; Itpr3, inositol 1,4,5-trisphosphate receptor 3; Map3k5, mitogenactivated protein kinase kinase kinase 5; Mcl1, myeloid cell leukemia-1; Nfkbia, nuclear factor-kappa-B inhibitor alpha; Nfkb1, nuclear factor-kappa-B 1; Ngf, nerve growth factor; Nras, N-Ras; Parp4, poly(ADP-ribose) polymerase family member 4; Pdpk1, phosphoinositide dependent protein kinase 1; Pik3cb, phosphatidylinositol-4,5-bisphosphate 3-kinase catalytic subunit beta; Pik3cd, phosphoinositide-3-kinase catalytic subunit delta; Pmaip1, phorbol-12-myristate-13-acetate-induced protein 1; Tnf, tumor necrosis factor; Tnfrsf1a, tumor necrosis factor receptor superfamily member 1 alpha; Tnfrsf1b, tumor necrosis factor receptor superfamily member 1 beta; Tnfrsf9, tumor necrosis factor receptor superfamily member 9; Tnfrsf11a, tumor necrosis factor receptor superfamily, member 11 alpha; Tnfrsf11b, tumor necrosis factor receptor superfamily members 11 beta; Tnfrsf12a, tumor necrosis factor receptor superfamily member 12 alpha; Tnfrsf13b, tumor necrosis factor receptor superfamily member 13 beta; Tnfrsf21, tumor necrosis factor receptor superfamily member 21; Tnfsf9, tumor necrosis factor superfamily member 9; Tnfsf13, tumor necrosis factor superfamily member 13; Tnfsf13b, tumor necrosis factor superfamily member 13 beta; Tnfsf15, tumor necrosis factor superfamily member 15; Tnfsf18, tumor necrosis factor superfamily member 18; Traf1, tumor necrosis factor receptor associated factor 1.

We have revealed that USP29 attenuates I/R-induced hepatic injury *in vitro* and *in vivo*. However, the exact mechanism by which USP29 exerts its function remained unclear. We performed RNA sequencing (RNA-Seq) using liver samples from WT and USP29-KO mice 3 hours after reperfusion. By principal component analysis (PCA), the expressed genes in the liver of WT and USP29 mice after I/R injury were clearly divided into two clusters (**Figure 5A**). In addition, GSEA analysis of GO showed that USP29 deficiency systematically activates inflammation and apoptosis-related pathways (**Figure 5B**). In addition, a heat map of GSEA-enriched pathway frontier subpopulations shows that apoptosis-related genes (**Figure 5C**), as well as inflammation-related genes (**Figure 5D**), were significantly increased in the USP29-KO group after hepatic I/R (relative to the same variables in the WT group). Our KEGG pathway enrichment analysis shows that in liver tissues undergoing hepatic I/R-mediated injury in the absence of USP29, mitogen-activated protein kinase (MAPK) was most significantly enriched (**Figure 5E**), suggesting that USP29 may protect from hepatic I/R injury through its effects on the MAPK pathway.

### USP29 inhibits TAK1-JNK/p38 pathway activation during hepatic I/R injury

**Figure 6:**
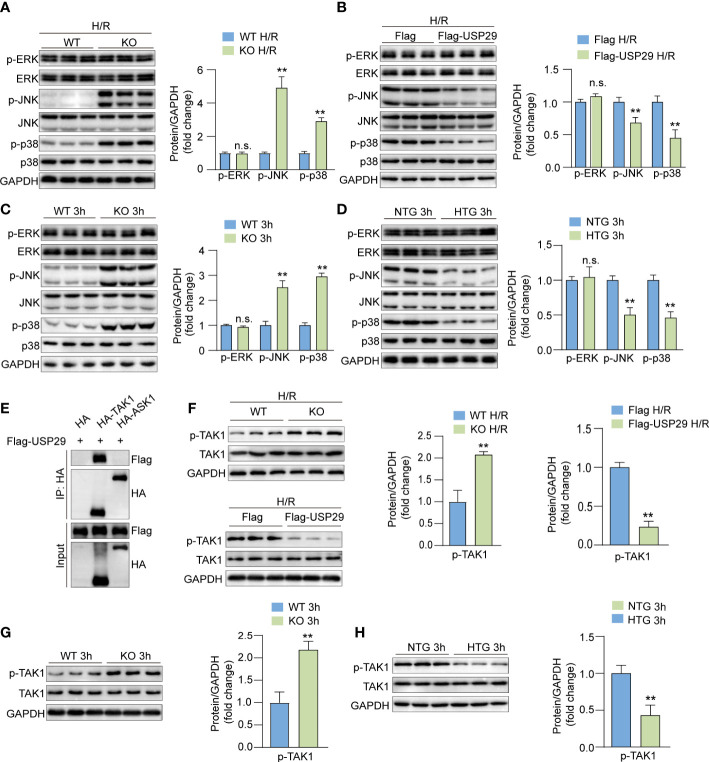
USP29 regulates TAK1-JNK/P38 pathway to reduce hepatic I/R injury. **(A)** Protein levels of total and phosphorylated ERK, JNK and p38 in primary hepatocytes from WT mice or USP29-KO mice treated with H/R (n = 3 per group). **(B)** Protein levels of total and phosphorylated ERK, JNK and p38 in primary mouse hepatocytes infected with AdUSP29 or AdVector treated with H/R (n = 3 per group). **(C)** Protein levels of total and phosphorylated ERK, JNK and p38 in livers of WT and USP29-KO mice with I/R injury (n = 3 per group). **(D)** Protein levels of total and phosphorylated ERK, JNK and p38 in livers of NTG and USP29-HTG mice with I/R injury (n = 3 per group). **(E)** Co-IP results of USP29 and TAK1 or ASK1. **(F)** Protein levels of total and phosphorylated TAK1 in primary hepatocytes from WT mice or and USP29-KO mice or infected with AdUSP29 and AdVector treated with H/R (n = 3 per group). **(G)** Detection of total and phosphorylated TAK1 in livers of WT mice and USP29-KO mice with I/R injury (n = 3 per group). **(H)** Detection of total and phosphorylated TAK1 in livers of NTG mice and USP29-HTG mice with I/R injury (n = 3 per group). GAPDH served as the loading control in **(A-D, F-H)**. All data are expressed as the means ± SD. n.s. indicates non significance between the two indicated groups; **p < 0.01. Ad, adenovirus; ASK1, apoptosis signal-regulating kinase 1; ERK, extracellular signal-regulated kinase; GAPDH, glyceraldehyde-3-phosphate dehydrogenase; IP, immunoprecipitation; p-ASK1, phosphorylated ASK1; p-ERK, phosphorylated ERK; p-JNK, phosphorylated JNK; p-p38, phosphorylated p38; p-TAK1, phosphorylated TAK1; TAK1, TGF-beta-activated kinase 1.

Next, we evaluated the USP29 effects on MAPK signaling. In primary mouse hepatocyte injury induced by H/R *in vitro*, USP29 deficiency promoted phosphorylation of JNK and p38 compared to control cells (**Figure 6A**), whereas USP29 overexpression inhibited phosphorylation of JNK and p38, ERK phosphorylation was not affected by the USP29 expression (**Figure 6B**). Consistent with our *in vitro* experiments results, in the livers of USP29-KO group mice subjected to I/R injury, we discovered that the USP29 KO promotes I/R-induced phosphorylation of JNK and p38but does not affect ERK phosphorylation relative to WT group (**Figure 6C**). Conversely, in the livers of I/R injury-induced USP29-HTG mice, the phosphorylation levels of JNK and p38, but not the ERK, was significantly inhibited (**Figure 6D**). TAK1 and ASK1 are MAPK family kinases that primarily activate downstream JNK/p38 signaling (19). Previous studies have shown that targeted inhibition of ASK1 and TAK1 can alleviate liver ischemia-reperfusion injury (20–22). As a result, we investigated which of the TAK1 and ASK1 USP29 proteins regulates the downstream JNK/p38 signaling pathway. Importantly, we found that USP29 interacted with TAK1 but not with ASK1 (**Figure 6E**). Moreover, in H/R-stimulated hepatocytes, phosphorylated TAK1 was significantly elevated in the absence of USP29 and significantly decreased in the USP29 overexpressing mice (**Figure 6F**). Furthermore, the effects of USP29 knockout or overexpression on TAK1 activation in livers undergoing hepatic I/R were consistent with the results we observed *in vitro* (**Figures 6G, H**). Taken together, these results suggest that USP29 inhibits the activation of the TAK1-JNK/p38 pathway during hepatic I/R injury.

### USP29 interacts with TAK1 and inhibits its K63-linked polyubiquitination and activation

**Figure 7:**
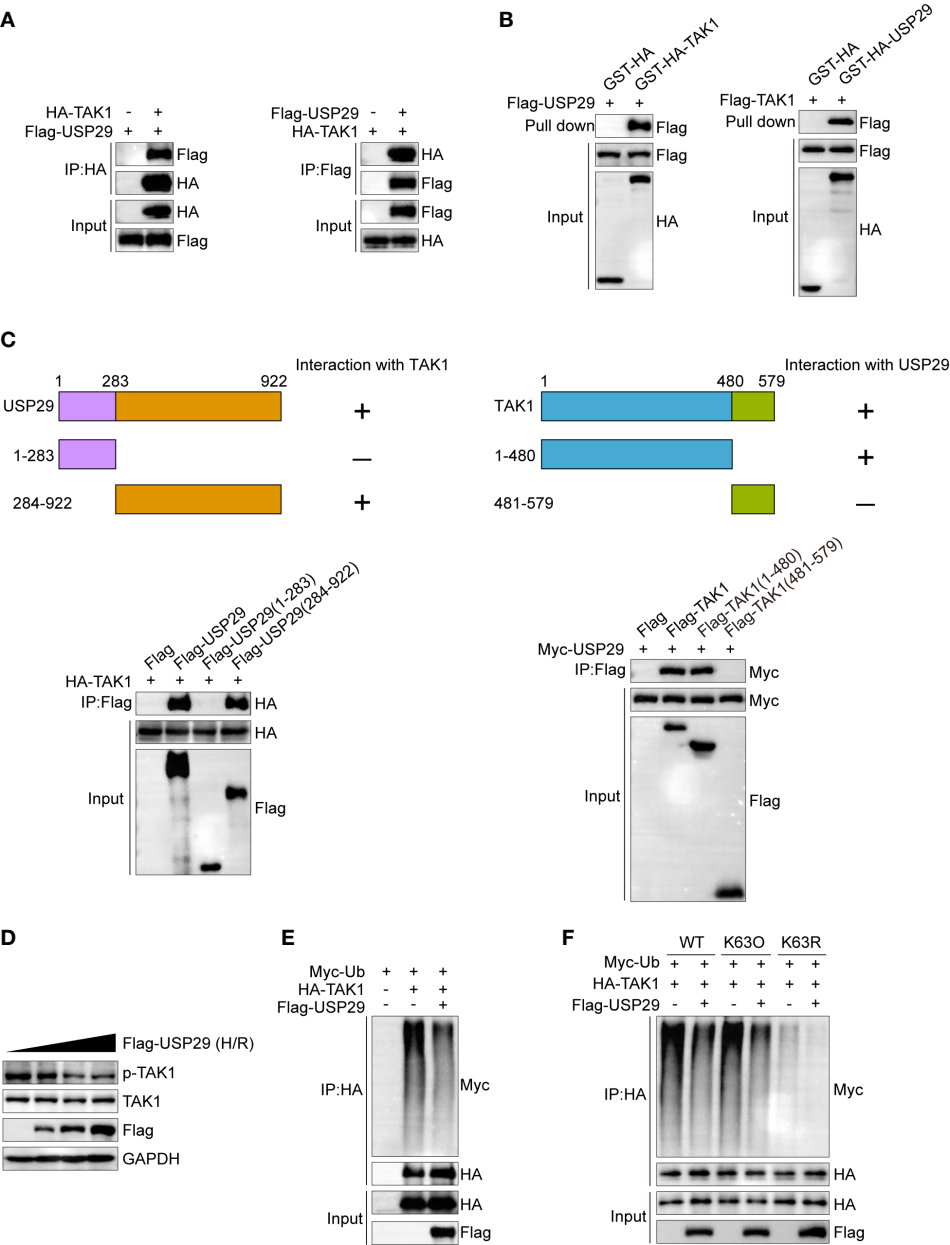
USP29 inhibits phosphorylation activation of TAK1 by inhibiting its K63 position ubiquitination. **(A)** Representative co-IP image of USP29 and TAK1 interaction. The FLAG-USP29 and HA-TAK1 plasmids were co-transfected into 293T cells. Western blots were detected with anti-FLAG antibody (left) or anti-HA antibodies (right). **(B)** GST pull-down assay showing the direct interaction between USP29 and TAK1 in HEK293T cells. Purified GST was used as a control. **(C)** Full-length of HA-TAK1 and various truncated mutants of Flag-USP29 (left) or full-length of Myc-USP29 and various truncated mutants of Flag-TAK1 (right) were constructed and coexpressed in HEK293 cells. Co-IP assays results showing the interacting structural domains between USP29 and TAK1. **(D)** Protein levels of p-TAK1 and TAK1 in L02 cells transfected with increasing doses of Flag-USP29 (0.5, 1, and2 µg) plasmids after hypoxia and reoxygenation. GAPDH served as a loading control. **(E)** IP (with anti-HA agarose beads) and immunoblot analysis (with anti-Myc) of HEK293 cells transfected with plasmids encoding HA-TAK1,Flag-USP29 together for 24 h. **(F)** IP (with anti-HA agarose beads) and immunoblot analysis (with anti-Myc) of HEK293 cells transfected with plasmids encoding HA-TAK1,Flag-USP29(K63O), FLAG-USP29(K63R) together for 24 h. Data are representative of three independent assays. HA, hemagglutinin; IP , immunoprecipitation, K63O, only lysine at position 63 remained intact, while lysines at other positions were mutated to arginine. K63R, lysine at position 63 was mulated to arginine, and other positions remained intact. Myc-Ub, ubiquitin tagged with the myc epitope.

We set up experiments to determine how USP29 positively affects TAK1 signaling. After co-transfecting Flag-tagged USP29 and HA-tagged TAK1 plasmids into HEK-293T cells, we demonstrated the interactions between USP29 and TAK1 by immunoprecipitation (**Figure 7A**). Moreover, glutathione s-transferase (GST) precipitation assay further demonstrated a direct interaction between USP29 and TAK1 (**Figure 7B**). In addition, we constructed truncated forms of the sequences of USP29 and TAK1 and determined by Co-IP technology the exact regions where USP29 and TAK1 interact. Notably, the ubiquitin hydrolase structural domain at the C-terminus of USP29 (284-922) and the N-terminal region fragment of TAK1 (1-480) mediated the direct interaction between these two proteins (**Figure 7C**). Moreover, we found that a dose-escalating USP29 expression effectively inhibited the TAK1 phosphorylation level (**Figure 7D**). USP29 is a deubiquitinating enzyme that mediates post-translational modifications of target proteins through deubiquitination (9). We investigated whether USP29’s regulatory effect on TAK1 is dependent on its deubiquitinating enzyme activity. As we expected, USP29 significantly reduced the level of ubiquitination of the TAK1 level (**Figure 7E**). K63-linked ubiquitination is a proteasome-independent modification that plays an important role in NF-κB activation by promoting TAK1 stability (23). Thus, we used K-63O and K-63R ubiquitin mutants and found that mutating lysine at the 63 site inhibited the TAK1 ubiquitination and that USP29 was able to significantly reduce the K63-linked TAK1 ubiquitination level (**Figure 7F**). These results suggest that USP29 interacts directly with TAK1 and inhibits its activation by removing a k63-linked ubiquitination signal.

### USP29 exerts its function by regulating the TAK1-JNK/p38 signaling pathway

**Figure 8:**
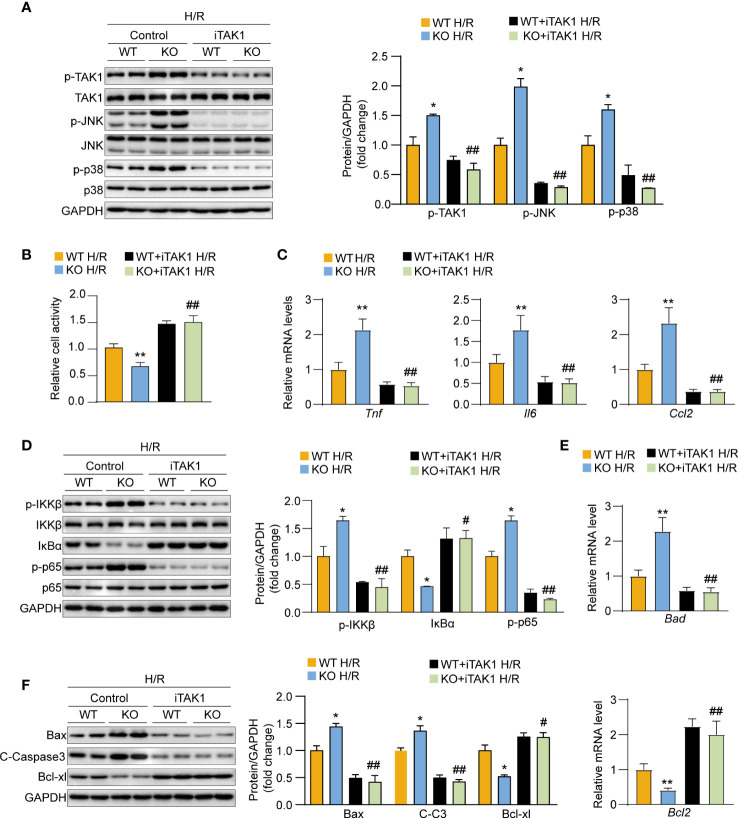
The exacerbation of hypoxia/reperfusion (H/R) damage after USP29 knockout depends on the effects of TAK1 in hepatocytes. **(A)** Western blot detection of total and phosphorylated protein expression levels of JNK, p38, TAK1 in primary hepatocytes from WT mice or USP29-KO mice treated with DMSO or TAK1 inhibitor (5Z-7-Ox) before H/R challenge. GAPDH served as the loading control (n = 3 per group). **(B)** Cell viability in in primary hepatocytes from WT mice or USP29-KO mice treated with DMSO or TAK1 inhibitor (5Z-7-Ox) subjected to H/R challenge. **(C)** mRNA levels of proinflammatory factors (Tnf, IL6, and Ccl2) in the indicated groups treated with H/R. **(D)** Protein levels of the NF-κB signaling components in the indicated groups treated with H/R detected by Western Blotting. **(E)** mRNA level of apoptosis-associated genes in the indicated groups treated with H/R. **(F)** Apoptosis-associated protein levels in the indicated groups treated with H/R. All data are expressed as the means ± SD. n.s. indicates non significance between the two indicated groups; *p < 0.05, **p < 0.01, USP29-KO H/R group compared with WT H/R group; #p < 0.1, ##p < 0.01, USP29-KO + iTAK1 H/R group compared with WT+ iTAK1 H/R group. C-Caspase3, cleaved caspase-3; Ccl2, C-C motif chemokine ligand 2; GAPDH, glyceraldehyde-3-phosphate dehydrogenase; IL-6, interleukin-6; IκBα, inhibitory κB α; IKKβ, IκB kinase β; iTAK1, TAK1 inhibitor; p-IKKβ, phosphorylated IKKβ; p-JNK, phosphorylated JNK; p-p38, phosphorylated p38;p-p65, phosphorylated p65; p-TAK1, phosphorylated TAK1; TAK1, TGF-beta-activated kinase 1; TNF, Tumor necrosis factor.

We used the TAK1-specific inhibitor 5Z-7-oxozeaenol (5Z-7-Ox) to block TAK1 activity in hepatocytes and thereby assess whether the role of USP29 in hepatic I/R injury is TAK1 dependent. We found that USP29 knockout significantly promoted TAK1-JNK/p38 pathway activation after the H/R challenge However, this effect was countered by blocking TAK1 activation (**Figure 8A**). Moreover, TAK1 inhibition during H/R stimulation was able to restore cell viability that had been depleted due to USP29 knockout (**Figure 8B**). Similarly, TAK1 inhibition significantly protected hepatocytes from USP29 silencing-induced inflammatory factors secretion, NF-κB signaling activation, and apoptosis-related factors increase (**Figures 8C–F**). Taken together, these results suggest that the role of TAK1 is necessary for USP29 protection against hepatic I/R injury.

## Discussions

Hepatic I/R injury is an important cause of liver injury during surgical operations; it can lead to liver failure in severe cases, largely hindering the salvage effect of liver surgery and negatively affecting the prognosis of patients (24). In this investigation, we found USP29 to be a viable target for the therapy of hepatic I/R injury. We discovered that USP29 expression was downregulated in both *in vivo* hepatic I/R injury and *in vitro* hepatocyte H/R models. Moreover, through USP29 function-gain and function-loss assays, we discovered USP29 has a protective role in hepatic I/R injury by attenuating the inflammatory response and apoptosis. We found that USP29 deregulated the polyubiquitinated state of the k63 TAK1 linkage through deubiquitination, thereby inhibiting TAK1 autophosphorylation and suppressing the activation of the downstream JNK/p38 pathway. TAK1 inhibitors eliminated the detrimental effects of USP29 knockout on mouse primary hepatocytes under H/R injury. These results suggest that USP29 protects against hepatic I/R injury by inhibiting the TAK1-JNK/p38 signaling pathway activation; we believe USP29 is a promising therapeutic target for hepatic I/R injury.

Hepatic I/R injury occurs in two phases: ischemia and reperfusion. Damage-associated molecular patterns (DAMPs) activate immune cells such as Kupffer cells and neutrophils; then, under the regulation of nuclear factor kappa B (NF-κB), pro-inflammatory factors such as *Tnf*, *Il1b*, adhesion molecules, and inducible nitric oxide synthase (iNOS) are activated and expressed to initiate and amplify the inflammatory response, resulting in apoptosis and necrosis of hepatocytes and causing liver injury (25–27). Numerous studies have confirmed that the over-activation of inflammatory signaling pathways and apoptosis can effectively inhibit hepatic I/R injury through inflammatory signaling pathways regulation. In our study, USP29 overexpression attenuated CD11b-positive inflammatory cells infiltration and downregulated expression of inflammatory factors *Tnf* and *Il1b* and pro-apoptotic factors Bax and Bad. In contrast, USP29 deficiency promoted CD11b-positive inflammatory cell recruitment (leading to increased expression of inflammatory and apoptotic factors) and significantly decreased expression of *Bcl2*, which inhibits mitochondrial apoptosis. Thus, our results suggest that USP29 attenuates hepatic I/R injury by inhibiting the inflammatory response and apoptosis.

We performed an RNA-sequencing (RNA-Seq) analysis of liver tissues from USP29 knockout mice and controls with I/R injury after 3h, and the results of the KEGG signaling pathway enrichment analysis showed that the MAPK signaling pathway was the most affected after USP29 knockout. The MAPK pathway mediates cellular responsesand is widely involved in cell growth and reproduction, division and death, and changes in various biochemical responses within cells. The conventional MAPK signaling pathway consists mainly of p38 (α, β, γ, and δ), c-jun amino-terminal kinases 1 to 3 (JNK1-3), extracellular signal-regulated kinases 1/2 (ERK1/2), and the ERK5 family (28). The MAPK signaling pathway is involved in I/R injury in various organs, as evidenced by studies on its three family members: JNK, ERK, and p38 MAPK (29–31). In our study, the JNK/p38 signaling pathway phosphorylation level was significantly reduced after I/R or H/R injury in USP29-overexpressing liver and hepatocytes (respectively), whereas USP29 knockout showed the opposite trend. The ERK expression and its phosphorylation levels were unaffected. Apoptosis signal-regulated kinase 1 (ASK1) and human transforming growth factor kinase 1 (TAK1) belong to the MAPKKK family and are located upstream of JNK and p38 in the MAPK signaling pathway: their activation can directly activate MAPKK and further activate JNK and p38 to cause inflammation and apoptosis (28). Our study shows that TAK1, but not ASK1, is the kinase that mainly activates downstream JNK/p38 signaling. USP29 regulates the TAK1-JNK/p38 signaling pathway and alleviates hepatic I/R injury.

USP29 functions as a typical ubiquitin proteasome family member through deubiquitination. USP29 inhibits ubiquitin-mediated proteasomal degradation of the Cdc25A protein by removing its polyubiquitination signal (10).USP29 directly deubiquitinates and stabilizes HIF1α, promotes its transcriptional activityand causes sorafenib resistance in hepatocellular carcinoma cells by increasing aerobic glycolysis (14). In addition, USP29 inhibits NF-κB activation and subsequent IFN-1 production during SARS-CoV-2 virus infection by deubiquitinating ORF9b, thereby enhancing the pathogen’s virulence (32). Therefore, we designed experiments to elucidate the mechanism by which USP29 regulates the TAK1-JNK/p38 signaling pathway. Further results demonstrated that USP29 directly bound and interacted with TAK1. Previous research has revealed that TAK1 autophosphorylation activation is dependent on its polyubiquitination (33, 34)and that numerous USP family members regulate TAK1 activity by targeting deubiquitination signals. For example, USP19 inhibits Tnf and Il1b-triggered NF-κB activation by targeting TAK1 uncoupling of k27 and k63-linked polyubiquitin chains (35). Moreover, the interaction of USP4 with TAK1 and the following TAK1 deubiquitination plays a key inhibitory role in non-alcoholic fatty liver disease (NAFLD) (36). USP18 binds and deubiquitinates TAK1 to attenuate hepatic steatosis and insulin resistance in non-alcoholic liver disease (37). Therefore, we speculate that USP29 may also regulate the activation of phosphorylation of TAK1 by regulating the deubiquitination of TAK1. As we expected, USP29 significantly decreased the level of ubiquitination of TAK1 after interacting with TAK1. The k48-linked polyubiquitination is mainly associated with proteasome-mediated degradation, whereas the k63-linked polyubiquitination usually regulates signal transduction (38). Phosphorylation activation of TAK1 is directly regulated by its K63-linked polyubiquitination (39). Consistently, USP29 regulates TAK1 activity and inhibits its autophosphorylation activation by removing the k63-linked polyubiquitin chain. Moreover, TAK1 inhibitors abrogated the protective effect of USP29 on hepatic I/R injury, suggesting that the regulation of hepatic I/R injury by USP29 is dependent on the TAK1-JNK/p38 pathway.

We used USP29 full knockout mice rather than hepatocyte-specific knockout mice, which may contain confounding effects of USP29 function in hepatic inflammatory cells. However, in general, our findings suggest that USP29 can protect the liver from hepatic I/R injury by removing the TAK1 polyubiquitination signal through deubiquitination, thereby inhibiting the TAK1-JNK/p38 pathway activation and leading to attenuation of the inflammatory response and apoptosis. Thus, our findings provide a new therapeutic target for preventing hepatic I/R injury.

## Data availability statement

The data presented in the study are deposited in the NCBI SRA repository, accession number PRJNA972855.

## Ethics statement

The studies involving human participants were reviewed and approved by Ethics Committee of Renmin Hospital of Wuhan University. The patients/participants provided their written informed consent to participate in this study. The animal study was reviewed and approved by Experimental Animal Welfare Ethics Review Committee of Renmin Hospital of Wuhan University.

## Author contributions

ZC, FH, YZ, LZ and TQ participated in the research design. ZC, YZ, LZ, TW, CK, HH, JG, QC, BY, YL and JLZ conducted the experiments and performed data analysis. ZC, FH, YZ and LZ wrote the manuscript. JQZ and TQ revised the manuscript and supervised the study. All authors contributed to the article and approved the submitted version.
