# Supplementary material for: Ubiquitin-specific protease 29 attenuates hepatic ischemia-reperfusion injury by mediating TGF-β-activated kinase 1 deubiquitination
Source: Front Immunol. 2023 May 26;14:1167667. doi: 10.3389/fimmu.2023.1167667 (PMC10250730; doi:10.3389/fimmu.2023.1167667)
Supplement: Supplementary file 1 [file DataSheet_1.docx]

**Supplementary Materials and Methods**

**Animals and treatment**

Male C57BL/6 mice, 8 weeks old, were purchased from Beijing Vital River Laboratory Animal Technology Co., Ltd. (Beijiing, China)  and housed in the Experimental Animal Center of the People's Hospital of Wuhan University. All mice were housed at 21°C±1°C, 55±10% humidity, 12 h of light/12 h of darkness, and food and water were provided ad libitum to the animals. All animal husbandry and experimental operations followed the laboratory management and ethical requirements of the People's Hospital of Wuhan University and were performed according to the guidelines of the Guide for the Care and Use of Laboratory Animals published by the National Institutes of Health.

The 70% hepatic ischemia-reperfusion (I/R) injury mouse model was constructed on 8-10-week-old male mice weighing 24-27 g according to the method described in our previous literature ^[1]^: The mice were anesthetized by intraperitoneal injection of 3% sodium pentobarbital (80 µg/g), abdominal and thoracic hair was removed with a shaver, the skin preparation site was disinfected with vital iodine and 75% alcohol three times, a longitudinal incision was made in the middle of the epigastrium into the abdomen, the abdominal organs were exposed to locate the left and middle lobes of the liver. The lower hepatic lobe was separated from the left and middle lobes with a small cotton ball, and the first hepatic hilar was fully exposedand released to gain access to the portal branches of the left and middle lobes of the liver. Non-invasive vascular clips were used to block blood flow to the middle and left lobes, causing 70% liver ischemia, but allowing blood to flow the right hepatic lobe to prevent portal vein and gastrointestinal tract stasis. At this point, the color of the left and middle lobes of the liver clearly changed from bright red to pale yellow. The abdominal cavity was closed with simple sutures. Mice in the Sham group underwent all the same surgical steps except for the clamping of the hepatic vessels. One hour after inducing the ischemia, the vascular clamps in the I/R mice were released and the abdomen was closed with layered sutures. The body temperature of the mice was kept constant during the whole procedure with the help of a monitor. According to the experimental design, mice were executed at 3 h, 6 h and 24 h after reperfusion, and serum, pathological and phlebotomy samples were collected.

USP29-knockout mice and USP29-transgenic mice were obtained according to the previously described method ^[2]^. Generate USP29 knockout mouse models using the CRISPR/ Cas9 system. The sequence-guide RNA of 2 target DNA regions was predicted on exon 9 of the *USP29* gene using an online CRISPR design tool (http://chopchop.cbu.uib.no/). pUC57-sgRNA (Addgene, 51132) was used as a backbone vector to construct the USP29-sgRNA expression vector. After mixing the purified product of *Cas9* mRNA and sgRNA, the mixture was injected into the single-cell fertilized eggs of C57BL/6 mice using a FemtoJet 5247 microinjection system, and the injected fertilized eggs were transplanted into surrogate females to obtain F0 generation mice after about 19–21 days of gestation. Toes tissues of mice were removed 1-2 weeks after birth, and genomic DNA was extracted for genetic identification. The Guidance sequences of USP29-KO mice are shown in the **Supplementary Table 1.**

To obtain USP29 transgenic (USP29-TG) mice, mouse cDNA obtained by reverse transcription was used as a template to amplify the coding region (CDS region) of the gene *USP29* and construct a pALB overexpression vector. The correctly sequenced plasmids were linearized by *PvuI* restriction endonuclease, and the recovered fragments were purified and used for microinjection to obtain F0 generation mice. Positive first build mice were then mated with wild type and screened to obtain F1 generation positive mice for breeding and line building, resulting in a stably inherited liver-specific USP29 transgenic mouse strain.

**Liver injury Measurements**

Mice in the sham-operated and I/R groups were anesthetized with 3% pentobarbital sodium at the indicated postoperative times. Blood was collected from the orbital venous plexus, serum was separated and diluted 15-fold with saline in the I/R group and undiluted in the Sham group, and serum glutathione aminotransferase (ALT) and glutathione aminotransferase (AST) levels were measured using an ADVIA 2400 biochemical analyzer (Siemens, Tarrytown, NY, USA). The left liver lobe of the ischemic area was frozen in liquid nitrogen or fixed in 10% formalin for 24 hours, then dehydrated and embedded into paraffin for sectioning.

**Pathological analysis**

**H&E staining**

The livers were removed and fixed in 10% formaldehyde solution for 72 h. After conventional dehydration and embedding, the largest cross-sectional sections were serially sectioned (5-μm slices), dewaxed, hydrated, stained, dehydrated, HE stained (Hematoxylin, G1004, Servicebio, Wuhan, China; Eosin, BA-4024, Baso, Zhuhai, China), sealed and observed under the microscope. Necrotic areas were analyzed and quantified using the Image Pro Plus software. For each mouse, the percentage of necrotic areas in the total area of tissue sections was quantified in more than five fields of view.

**Immunofluorescence**

Paraffin liver sections were dewaxed and hydrated, then stained for CD11b and TUNEL.

CD11b: First, EDTA high-temperature repair was performed for 20 min, then mouse liver sections were blocked with 10% BSA, followed by incubation with primary antibody (Boster, BM3925) at 4 °C overnight. The sections were then washed with PBS and incubated with secondary antibody (Alexa Flour® 568 goat anti-Rabbit IgG(H+L) A11036, Invitrogen, Carlsbad, CA, USA) at 37 °C for 1 h. Nuclei were stained with DAPI.

TUNEL: Staining was performed using the TUNEL staining kit (Roche, 11684817910), according to manufacturer’s instructions.

**Immunohistochemical staining**

Paraffin liver sections were dewaxed and hydrated before staining them for USP29 immunohistochemistry. The sections were first repaired with EDTA at high temperature for 20 min, then blocked with 10% BSA, followed by incubation with primary antibody (USP29, Proteintech, 27522-1-AP) overnight at 4 °C and rewarmed at 37 °C for 30 min. Next, the sections were washed with PBS and incubated with secondary antibody (Rabbit Two-step Detection Kit, PV-9001, ZSGB-BIO, Beijing, China). After completion of the incubation, the slices were stained with DAB (ZLI-9018, ZSGB-BIO, Beijing, China) for 1 min, hematoxylin lined, and sealed.

**Cell culture**

L02 and HEK293 cells were purchased from the Typical Culture Collection Committee Cell Bank, Chinese Academy of Sciences (Shanghai, China). HEK293 cells and primary hepatocytes were cultured in DMEM containing 10% fetal bovine serum (F05-001-B160216; Bio-One Biotechnology, Guangzhou, China) and 1% penicillin-streptomycin (15140-122; Gibco by Invitrogen), at 37° C with 5% CO2 in an incubator. To inhibit TAK1 activation, hepatocytes were treated with a TAK1 inhibitor (5Z-7-Oxozeaenol) (O9890-1 MG, Sigma, St. Louis, MO, USA) at a concentration of 2 µM.

**Isolation of primary hepatocytes**

Mouse primary hepatocytes were isolated from 6 to 8-week-old male mice using the type IV collagenase(17104019; GIBCO, USA) digestion method^[3]^. After anesthetizing the mice, the abdominal cavity was exposed by opening the abdomen, an indwelling needle was placed in the inferior vena cava, liver lavage solution was instilled at a constant temperature of 37 °C. After moderately filling the liver, the portal vein was cut and the liver was fully perfused to rinse off the blood. The lavage solution was replaced with lavage solution containing type IV collagenase for digestion, and after digestion, the whole liver was removed and the liver envelope was torn open to free the hepatocytes. Cells were harvested by passing the suspension through a 70 µm strainer(352350; BD, USA) and centrifuged at 4℃ at 50g for 3 min.

**Cellular hypoxia–reoxygenation model**

Isolated hepatocytes were plated on collagen (354236; BD, USA) coated dishes and cultured for 24 h. Then the cells were washed, and the medium was replaced with sugar-free serum-free medium in a triple-gas incubator at 1% O_2_, 5% CO_2_, 94% N_2_, and 37°C for 1 h. Next, the medium was replaced with high-pond medium containing 10% fetal bovine serum and reoxygenated at 37°C for 6 h in a 5% CO_2_ incubator.

**RNA-seq and DEG analysis**

The single-end library was sequenced using BGISEQ 500 (UWIC) with a read length of 50 bp. The sequenced fragments were aligned to the mouse reference genome (mm10/GRCm38) using the HISAT2 software (version 2.1.0), and the files obtained were then converted using SAMtools (version 1.4) to binary BAM format to store the matching information. Next, the exon model fragment per kilobase fragment per million (FPKM) values were calculated for each identified gene using the default StringTie (version 1.3.3b) parameters. Then, DESeq2 (version 1.2.10) identified differentially-expressed genes (DEGs) based on the following two criteria: (1) ploidy change greater than 2; (2) corresponding *p*-value lower than 0.05. Finally, the ggplot2 package was used to make volcano plots showing the differential ploidy and *p*-value for all genes.

**Hierarchical clustering**

Hierarchical clustering creates a hierarchical nested clustering tree by calculating the similarity between different samples using a weighted average distance (weighted two-group arithmetic method, UPGMA) algorithm, which was then visualized with the hclust function of the R package.

**KEGG and Gene Ontology Analyses**

KEGG (Kyoto Encyclopedia of Genes and Genomes) is a comprehensive database that integrates genomic, chemical, and phylogenetic functional information. KEGG pathway enrichment analysis was performed for all differential genes by Fisher's exact test, and KEGG pathway annotations for all genes in the reference genome were downloaded from the KEGG database. The pathways with *p*-value lower than 0.05 were defined as significantly enriched. The signaling pathways were sorted by the number of differential genes enriched in each pathway from highest to lowest and visualized by R package ggplot2.

**Gene Set Enrichment Analysis (GSEA)**

Gene Set Enrichment Analysis (GSEA) is performed by sorting genes by differential expression using gene sets in the KEGG and GO pathways, sequentially, and then examining whether the gene sets are clustered at the top or bottom of this ranking table to obtain the overall expression changes of gene sets. The analysis was performed on the Java GSEA (version 3.0) platform using the "Signal2Noise" metric, and gene sets with *p*-values lower than 0.05 and FDR lower than 0.25 were considered statistically significant.

**Real-time quantitative PCR:**

Total RNA was extracted from cultured hepatocytes and hepatic tissues using TRIzol reagent (T9424, Sigma) according to the manufacturer’s instructions. After transcribing RNA into cDNA using a Transcriptor First Strand cDNA Synthesis Kit (R323-01, Vazyme), quantitative real-time PCR (*q*RT-PCR) amplification was performed using SYBR Green PCR Master Mix (Q111-02, Vazyme). The PCR program was set at 95 °C for 5 min, followed by 40 cycles at 95 °C for 15 s and 60 °C for 60 s. The relative mRNA expression levels were calculated using the 2^−ΔΔCt^ method and were normalized against β-actin. All the primers are listed in the **Supplementary Table 3**.

**Cell counting kit-8 assay:**

Cell Counting Kit-8 (CCK-8) assays were performed according to the manufacturer’s instructions (44786; Dojindo, Wuhan, China). Hepatocytes were seeded into 96-well plates (167008; Thermo Fisher Scientific, Carlsbad, CA, USA) at a density of 5000 cells per well. Cells were treated with H/R after cell attachment, and then Cells were incubated for 2 h in the 37 °C incubator. Cell absorptivity was measured at a wavelength of 450 nm.

**Plasmids construction**

The plasmids were constructed as previously described^[4]^. The full-length CDS sequences of USP29, TAK1 and ASK1 were amplified from human cDNA, and the full-length CDS of USP29, TAK1, and ASK1 were concatenated into pcDNA5-flag, pcDNA5-HA, and GST-HA vectors using In-Fusion recombination to obtain USP29, TAK,1 and ASK1 full-length overexpression plasmids (Flag tag, HA tag, and GST-HA tag, respectively). We also designed primers to construct truncated plasmids for Flag-USP29 (1-283, 284-922) and Flag-TAK1 (1-480, 481-579).

**Adenovirus constructs**

The CDS sequence of USP29 was subcloned into the shuttle vector pENTR- CMV-flag-T2A-GFP to create a mouse USP29 overexpression adenovirus. The plasmid described above was reconstituted with pAdeasy as the backbone vector, PacI linearized, and transfected into 293A cells. The Adeasy Adenovirus Packaging System was used to obtain adenovirus. As a control, the gene encoding GFP (AdGFP) was used. The titer of recombinant adenovirus was determined to be 10^10^ pfu/ml. The prisms used to construction of mouse USP29 overexpression adenovirus are shown in the **Supplementary Table 1.**

**Western blotting analysis**

Proteins were extracted from ischemic liver tissue or cell lysates, and the total concentration was determined using the BCA (23225, Thermo) method. Protein samples of the same mass were added to loading buffer and separated by 10% SDS-PAGE. After electrophoresis, the proteins were transferred onto 0.45 μm PVDF (IPVH00010, Millipore) membranes. PVDF membranes were blocked with 5% skim milk powder containing 0.1% Tween 20 (TBST) for approximately 1 hour at room temperature. The PVDF membrane was washed three times (5 min each) with TBST, and then incubated with primary antibody overnight at 4 °C. The cells were washed three times with TBST and incubated with peroxidase-conjugated secondary antibody (Cell Signaling Technology, Danvers, MA) for 1 h at room temperature. The final blots were visualized with signals from the Burroughs Gel Imaging System (ChemiDoc XRS, +). Antibodies used are shown in the **Supplementary Table 4**.

**Co-immunoprecipitation assays**

The desired plasmids were first co-transfected into 293T cells, and 24 h after transfection the cells were lysed with IP lysis buffer (20 mM Tris-HCl, pH 7.4; 150 mM NaCl; 1 mM EDTA and 1% NP-40). After high speed centrifugation at 4 °C, the protein-containing supernatant was incubated overnight with protein G agarose beads and the indicated anti-labeling IP antibody at 4 °C. The beads were centrifuged at 3000 rpm at 4 °C, washed 3 times with buffer containing 300 mM and 150 mM NaCl, and resuspended with 2X SDS loading buffer before being heated at 95 °C for 5–10 min and then analyzed in a Western blot assay.

**GST-Pull down**

GST-HA-TAK1 (or USP29) and Flag-USP29 (or TAK1) proteins were overexpressed in 293T cells, and cells were lysed using lysis solution (50 mM Na2HPO4, pH 8.0; 300 mM NaCl; 1% TritonX-100). GST-HA-TAK1 (or USP29) protein was purified using GST beads, and GST beads were de-hybridized from Flag-USP29 (or TAK1) protein. GST-HA-TAK1 (or USP29) and Flag-USP29 (or TAK1) proteins were mixed and incubated overnight at 4 °C. The beads were washed 3 times with buffer (20 mM; 150 mM NaCl; 0.2% TritonX-100), resuspended with 2X SDS loading buffer and heated at 95 °C for 5–10 min, before Western blot analysis.

**Ubiquitination assays**

The desired plasmids were first co-transfected into 293T cells, and 24 h after transfection each sample was lysed with 100 μL of 10% SDS lysis buffer and denatured by heating at 95 °C for 10 min. Next, 0.9 ml of pre-cooled IP lysis buffer (20 mM Tris-HCl, pH 7.4; 150 mM NaCl; 1 mM EDTA and 1% NP-40) were added to this lysis product. After sonication, the samples were centrifuged (12000 *g*, 10 min). The supernatant was then extracted and incubated with primary antibody and protein A/G agarose beads (11719394001,11719386001; Roche; Basel, BS, Switzerland) for 3 h at 4 °C. The beads were then washed five times with IP lysis buffer and then boiled with SDS loading buffer for 10 min to elute the proteins before Western blot analysis.

**Supplementary Table 1.** Guidance sequences of USP29-KO mice and primers used to construction of mouse USP29 overexpression adenovirus.

| **Primer** | **Sequence 5’-3’** |
| --- | --- |
| USP29-sgRNA 1 | TGATAATGTTACAGGCGTAG TGG |
| USP29-sgRNA 2 | AGTGGTATTCAGGGATCCAC AGG |
| USP29-P1 | GAACCAATGAAATGCGAGGT |
| USP29-P2 | AGTAGACGAATCCAGGAATG |
| Ad USP29-mouse-F: | GCTAGCGATATCGGATCCGCCACCATGGCTCACCTAAAGATAAATGGTTTGG |
| Ad USP29-mouse-R: | ACTAGTGGTACCAAGCTTGAATTCTTCCAGGATGTATGTAAGGCC |

**Supplementary Table 2**. Primers for plasmid construction.

| **Gene** | **Species** |  | **Sequence 5’-3’** |
| --- | --- | --- | --- |
| Flag-*USP29* | Human | F | TCGGGTTTAAACGGATCCATGATATCTCTAAAGGTATGTGGATTCATCCA |
|  |  | R | GGGCCCTCTAGACTCGAGTCAAGCAGGTCTGTACAAAGAGTCACC |
| Flag-*USP29*(1-283) | Human | F | TCGGGTTTAAACGGATCCATGATATCTCTAAAGGTATGTGGATTCATCCA |
|  |  | R | GGGCCCTCTAGACTCGAGCAGTTGCTGTGAATGAGAGTCAAGAGG |
| Flag-*USP29*(284-922) | Human | F | TCGGGTTTAAACGGATCCCAGCAGGGGTTCCCCAATTTG |
|  |  | R | GGGCCCTCTAGACTCGAGTCAAGCAGGTCTGTACAAAGAGTCACC |
| HA-*TAK1* | Human | F | AGAGCCCGGGCGGATCCATGTCTACAGCCTCTGCCGC |
|  |  | R | TCGACGAATTGCTCGAGTCATGAAGTGCCTTGTCGTTTCT |
| HA-*ASK1* | Human | F | TCGGGTTTAAACGGATCCATGAGCACGGAGGCGGACG |
|  |  | R | GGGCCCTCTAGACTCGAGTCAAGTCTGTTTGTTTCGAAAG |
| *GST*-HA-*USP29* | Human | F | TCGGGTTTAAACGGATCCATGATATCTCTAAAGGTATGTGGATTCATCCA |
|  |  | R | GGGCCCTCTAGACTCGAGTCAAGCAGGTCTGTACAAAGAGTCACC |
| *GST*-HA-*TAK1* | Human | F | TCGGGTTTAAACGGATCCATGTCTACAGCCTCTGCCGCCT |
|  |  | R | GGGCCCTCTAGACTCGAGTCATGAAGTGCCTTGTCGTTTCT |
| Flag-*TAK1* | Human | F | TCGGGTTTAAACGGATCCATGTCTACAGCCT  CTGCCGCCT |
|  |  | R | GGGCCCTCTAGACTCGAGTCATGAAGTGCCT  TGTCGTTTCT |
| Flag-*TAK1*(1-480) | Human | F | AGAGCCCGGGCGGATCCATGTCTACAGCCTCTGCCGC |
|  |  | R | TCGACGAATTGCTCGAGTCATAGTTGGTGATCCAGTGTAAGATAAGC |
| Flag-*TAK1*(481-579) | Human | F | AGAGCCCGGGCGGATCCCTACAGCCTCTAGCACCGTG |
|  |  | R | TCGACGAATTGCTCGAGTCATGAAGTGCCTTGTCGTTTCT |
| Myc-*USP29* | Human | F | TCGGGTTTAAACGGATCCATGATATCTCTAAAGGTATGTGGATTCATCCA |
|  |  | R | GGGCCCTCTAGACTCGAGTCAAGCAGGTCTGTACAAAGAGTCACC |
|  |  |  |  |
|  |  |  |  |

**Supplementary Table 3.** Primers used in qRT-PCR.

| **Gene** | **Species** |  | **Sequence 5’-3’** |
| --- | --- | --- | --- |
| *TNF-α* | Mouse | F | CATCTTCTCAAAATTCGAGTGACAA |
|  |  | R | TGGGAGTAGACAAGGTACAACCC |
| *IL-6* | Mouse | F | TAGTCCTTCCTACCCCAATTTCC |
|  |  | R | TTGGTCCTTAGCCACTCCTTC |
| *IL-8* | Mouse | F | GGATCCTGATGCTCCATGGG |
|  |  | R | CAGAAGCTTCATTGCCGGTG |
| *IL-1β* | Mouse | F | CCGTGGACCTTCCAGGATGA |
|  |  | R | GGGAACGTCACACACCAGCA |
| *Ccl2* | Mouse | F | TACAAGAGGATCACCAGCAGC |
|  |  | R | ACCTTAGGGCAGATGCAGTT |
| *Ccl5* | Mouse | F | TGCTGCTTTGCCTACCTCTC |
|  |  | R | TCTTCTCTGGGTTGGCACAC |
| *Bad* | Mouse | F | CCAGAGTTTGAGCCGAGTGAGCA |
|  |  | R | ATAGCCCCTGCGCCTCCATGAT |
| *Bax* | Mouse | F | TGAGCGAGTGTCTCCGGCGAAT |
|  |  | R | GCACTTTAGTGCACAGGGCCTTG |
| *Bcl2* | Mouse | F | TGAGCGAGTGTCTCCGGCGAAT |
|  |  | R | GCACTTTAGTGCACAGGGCCTTG |
| *Bcl-xl* | Mouse | F | TTCAGCACGAGCAGTCAGCCAG |
|  |  | R | GCTCCCGGTTGCTCTGAGACATT |
| *β-actin* | Mouse | F | GTGACGTTGACATCCGTAAAGA |
|  |  | R | GCCGGACTCATCGTACTCC |

**Supplementary Table4.** List of antibodies.

| **Antibody** | **Cat No.** | **Manufacturer** |
| --- | --- | --- |
| USP29 | 27522-1-AP | Proteintech |
| GAPDH | 2118 | CST |
| p-Ikkβ | 2694 | CST |
| Ikkβ | A0714 | Abclonal |
| IkBα | 4814 | CST |
| p-p65 | 3033 | CST |
| p65 | 8242 | CST |
| Bad | 9292 | CST |
| Bax | A1964 | Abclonal |
| C-Caspase3 | 9664 | CST |
| Bcl2 | A19693 | Abclonal |
| p-ERK | 4370 | CST |
| ERK | 4695 | CST |
| p-JNK | 4668 | CST |
| JNK | 9252 | CST |
| p-p38 | 4511 | CST |
| p38 | 8690 | CST |
| TAK1 | Ab109526 | Abcam |
| p-TAK1 | 4508S | CST |
| Flag | M185-3LL | MBL |
| HA | M180-3 | MBL |
| Myc | M047-3 | MBL |

[1] Qiu T, Wang T, Zhou J, et al. DUSP12 protects against hepatic ischemia-reperfusion injury dependent on ASK1-JNK/p38 pathway in vitro and in vivo [J]. Clin Sci (Lond), 2020, 134(17): 2279-94.

[2] Jiangqiao Z, Tianyu W, Zhongbao C, et al. Ubiquitin-Specific Peptidase 10 Protects Against Hepatic Ischaemic/Reperfusion Injury via TAK1 Signalling [J]. Front Immunol, 2020, 11(506275.

[3] Tao Q, Tianyu W, Jiangqiao Z, et al. Tripartite Motif 8 Deficiency Relieves Hepatic Ischaemia/reperfusion Injury via TAK1-dependent Signalling Pathways [J]. International journal of biological sciences, 2019, 15(8): 1618-29.

[4] Zhou J, Guo L, Ma T, et al. N-acetylgalactosaminyltransferase-4 protects against hepatic ischemia/reperfusion injury by blocking apoptosis signal-regulating kinase 1 N-terminal dimerization [J]. Hepatology, 2022, 75(6): 1446-60.
